# Metabolites That Confirm Induction and Release of Dormancy Phases in Sweet Cherry Buds

**DOI:** 10.3390/metabo13020231

**Published:** 2023-02-03

**Authors:** Klaus-P. Götz, Frank-M. Chmielewski

**Affiliations:** Agricultural Climatology, Faculty of Life Sciences, Humboldt-University of Berlin, Albrecht-Thaer-Weg 5, 14195 Berlin, Germany

**Keywords:** *Prunus avium* L., cultivar Summit, dormancy phases, targeted metabolite profiling, chrysin, arabonic acid, pentose acid, sucrose, abscisic acid, abscisic acid glucose ester

## Abstract

Here we report on metabolites found in a targeted profiling of ‘Summit’ flower buds for nine years, which could be indicators for the timing of endodormancy release (t_1_) and beginning of ontogenetic development (t_1_*). Investigated metabolites included chrysin, arabonic acid, pentose acid, sucrose, abscisic acid (ABA), and abscisic acid glucose ester (ABA-GE). Chrysin and water content showed an almost parallel course between leaf fall and t_1_*. After ‘swollen bud’, water content raised from ~60 to ~80% at open cluster, while chrysin content decreased and lost its function as an acetylcholinesterase inhibitor. Both parameters can be suitable indicators for t_1_*. Arabonic acid showed a clear increase after t_1_*. Pentose acid would be a suitable metabolite to identify t_1_ and t_1_*, but would not allow describing the ecodormancy phase, because of its continuously low value during this time. Sucrose reached a maximum during ecodormancy and showed a significant correlation with air temperature, which confirms its cryoprotective role in this phase. The ABA content showed maximum values during endodormancy and decreased during ecodormancy, reaching 50% of its content t_1_ at t_1_*. It appears to be the key metabolite to define the ecodormancy phase. The ABA-GE was present at all stages and phases and was much higher than the ABA content and is a readily available storage pool in cherry buds.

## 1. Introduction

As a result of an untargeted global metabolite profiling to detect relevant metabolites which are related to the induction, maintenance, and release of dormancy phases, 445 named metabolites were found in sweet cherry buds [1]. An important aspect in the selection of suitable metabolites is the identification of candidates which are under environmental control and/or which have a proven physiological impact in the respective dormancy phase. Such metabolites would be of particular interest for phenological modelling because they would allow to model the non-observable stages of endodormancy release (t_1_), when the cultivar specific chilling requirement is fulfilled, and the beginning of ontogenetic development (t_1_*), the latter of which cannot be recorded by phenological observations and must start a few weeks before any visible changes in the buds are detectable [2]. 

For this reason, it was at first necessary to detect the timing of t_1_ and t_1_* before it was possible to identify any metabolites that might be related to the induction or release of winter dormancy. For nine years, we observed under controlled conditions the timing of t_1_ by means of twig samplings, and analyzed t_1_* according to changes in the flower bud’s water content in the orchard [2,3]. Identifying biomarkers that can reliably indicate the transition between phenological phases would substantially improve phenological modelling and could allow for developing physiologically justified model approaches, instead of statistical ones.

In untargeted metabolite profiling [1], chrysin, arabonic acid, pentose acid, sucrose, abscisic acid (ABA), and abscisic acid glucose ester (ABA-GE) (Figure S1), which are reported here, showed trend changes at the important stages, t_1_ and/or t_1_*. These metabolites which show an ‘u-shape’ or its inverse form between ‘leaf fall’ (LF) and ‘open cluster’ (OC) can be key markers, which allowed us to confirm the previously found dates for t_1_ and t_1_*. On one hand, it must be noted, the physiological role of chrysin, arabonic acid and pentose acid in plants has been described so far very little in the literature, and certainly not in cherry buds. Chrysin for example, is most known for its effect in humans (anti-inflammation, anti-cancer, anti-oxidation) [4,5,6,7,8]. It is a 5,7-dihydroxy-2-phenyl-4H-chromene-4-one or 5,7-dihydroxyflavone, a naturally 15-carbon backbone-based flavone compound assigned to the group of flavonoids, that are present in fruits and plants. These plant pigments are divided into flavones, flavanones, flavonols, anthocyanidins, flavanols, and isoflavones, according to the oxidation degree in the central C ring, the hydroxylation pattern of the rings, and the substitution at the 3 positions [9]. Again, the physiological function of chrysin in the plant kingdom has so far been only limited described.

On the other hand, it is well known that the phytohormone ABA plays an important role in plant development and is involved in response to stress, for example, temperature, water shortage, and drought stress activated ABA biosynthesis, transport and catabolism [10,11,12,13]. The compartmentalization of ABA and its catabolites, phaseic acid (PA) and dihydroxyphaseic acid (DPA), is important for ABA equilibrium, above all for ABA-GE, as it can provide ABA via a one-step hydrolysis and takes care in ABA homeostasis [14,15]. Accumulation of saccharides, e.g., sucrose, is reported for many plants, and correlates with the development of cold and freezing tolerance [16,17].

## 2. Materials and Methods

The description of the experimental site, phenological observations in the orchard, determination of ecodormancy phase (t_1_–t_1_*), sampling of sweet cherry buds and preparing for analysis has already been published in detail [1,2,3,18]. 

### 2.1. Experimental Site

The targeted metabolite profiling was conducted from flower bud samples for nine years (2011/12–2019/20) at Berlin-Dahlem (52.47° N, 13.30° E, altitude = 51 m a.s.l.). Trees were grafted on Gisela-5 rootstocks. All investigations in this study were focused on the ‘Summit’ cultivar, of origin from British Columbia. The long-term annual mean air temperature and precipitation (1991–2020) were 10.4 °C and 562 mm, respectively.

### 2.2. Sampling of Sweet Cherry Buds

In order to analyze chrysin, arabonic acid, pentose acid, sucrose, ABA, and the ABA-GE content in the flower buds, in each year between the LF and OC stages, three bud clusters of four (2011/12–2017/18) and three trees each (2018/19–2019/20) were taken weekly in the orchard at random locations over the whole tree. After the beginning of bud development sampling was done at the stages ‘swollen bud’ (SB), ‘side green’ (SG), ‘green tip’ (GT), ‘tight cluster’ (TC) and ‘open cluster’ (OC). After cutting, clusters were immediately placed in plastic bags on ice. They were consequently frozen in liquid nitrogen and stored at −80 °C until freeze-drying. All buds were ground in a ball mill (Retsch M1, Haan, Germany) before analysis. 

To determine the beginning of ontogenetic development (t_1_*), additional flower buds were sampled between September and April to calculate the bud’s water content. During ecodormancy, this value was almost constant at 54%, and only began to rise continuously at the end of winter, indicating the beginning of ontogenetic development. 

### 2.3. Targeted Metabolite Profiling 

The targeted metabolite profiling (chrysin, arabonic acid, pentose acid, sucrose, ABA, ABA-GE) was conducted by Metabolon Inc., 617 Davis Drive, Morrisville, NC 27560 (www.metabolon.com, accessed on 15 December 2022). Lyophilized sweet cherry bud samples were extracted with Methanol:Water (70:30) containing stable labelled or analog internal standards, and were subjected to homogenization. After centrifugation, an aliquot of the supernatant was removed and added to an equal volume of blank extraction solvent within a 96-well plate. After mixing, two aliquots of the extract diluent were transferred into separate 96-well plates; one was analyzed using a reverse phase method and a second plate underwent a derivatization step before analysis. The plates were injected on an Agilent 1290/AB Sciex QTrap 5500 LC-MS/MS system equipped with C18 reversed phase UHPLC column. The mass spectrometer was operated in positive and negative mode using Scheduled MRM™ for the reverse phase method and in negative mode for the derivatization method. In both methods ionization was achieved using heated electrospray ionization via Sciex’s TurboIonSpray^®^ source. 

The peak area of the individual analyte product ions was measured against the peak area of the product ions of the corresponding internal standards. Quantitation was performed using a weighted linear or quadratic least squares regression analysis generated from fortified calibration standards prepared immediately prior to each run. Additionally, qualitative analysis was performed on four pentose sugar acids with the same product ions as arabonic/xylonic acid by measuring the peak area of the pentose acid in the sample against the peak area of the corresponding internal standard used for quantitating arabonic/xylonic acid. Due to physiochemical homology, arabonic acid and xylonic acid were unable to be chromatographically resolved using authentic reference standards even after extensive method development work. Arabonic acid was selected as the reference standard for calibrators, however the reported result contains the contribution of both arabonic and xylonic acids. The LC-MS/MS raw data were collected and processed using AB SCIEX software Analyst 1.6.2 and processed using SCIEX OS-MQ software v1.7. Data reduction was performed using Microsoft Excel for Office 365 v.16.

### 2.4. Statistical Analysis 

Multiple comparison of means was performed with IBM SPSS V29 software, using one-way ANOVA with Tukey HSD-test. We used the two-sided test, testing whether the means are different at *p* ≤ 0.05. Figures were plotted with IGOR Pro V6.3.7.2.

## 3. Results and Discussion

Table 1 summarizes the mean metabolite content at five relevant phenological stages and in four phenological phases, including the endodormancy (LF–t_1_) and ecodormancy phase (t_1_–t_1_*). The phase of ontogenetic development was divided into two phases (t_1_*–SB, SB–OC), as some metabolites showed only distinct content-changes after the beginning of bud swelling, when the biological activity of the bud becomes visible. However, the induction of this process already starts on average 3 weeks earlier (22.3 ± 7.8 d), at t_1_*. Sucrose and ABA differ from chrysin, arabonic acid, pentose acid, and ABA-GE, in the respect that their content during ecodormancy (t_1_–t_1_*) shows significant differences compared to endodormancy and ontogenetic development, respectively.

### 3.1. Chrysin and the Water Content in Sweet Cherry Buds

**Figure 1:**
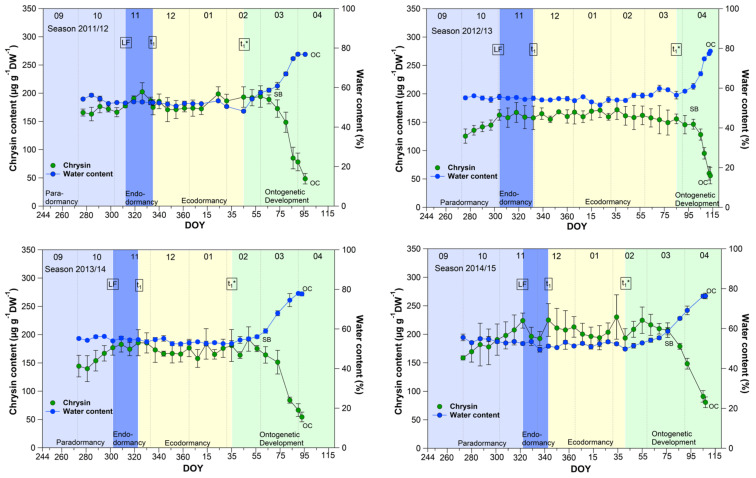

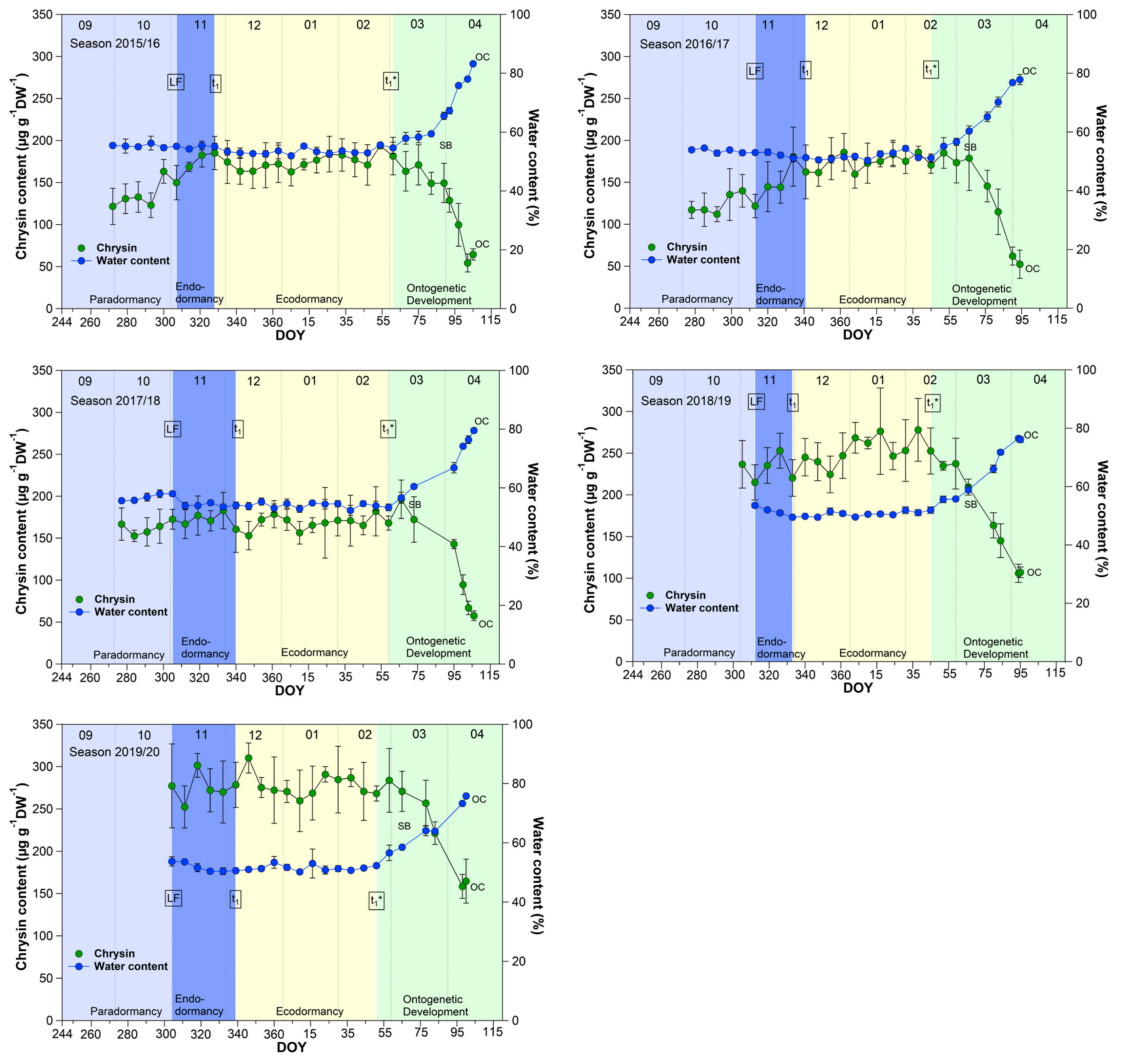
Chrysin and water content in sweet cherry buds cv. Summit, 2011/12–2019/20. ‘Total leaf fall’ (LF), endodormancy release (t_1_), beginning of ontogenetic development (t_1_*), ‘swollen bud’ (SB) and ‘open cluster’ (OC).

The course of the chrysin and water content in the buds (Figure 1) showed an almost parallel course over 9 years, between leaf fall (LF) and beginning of ontogenetic development (t_1_*). From the swollen bud stage (SB) on, the water content in the buds rose continuously from ~60% to ~80% at OC, while the chrysin content decreased significantly (*p* ≤ 0.05) in the opposite direction by ~60% from 185.7 μg/(g DW) to 76.2 μg/(g DW) at OC (Table 1). 

Water resorption and water homeostasis in plants is influenced by a “cholinergic” system [19], where acetylcholine (ACh), an ester of acetic acid and choline, is involved. Some examinations have been carried out on the effect of ACh on growth and development processes in plants. Acetylcholine may control plant growth through the regulation of membrane permeability as well the transport of storage substances to rapidly growing tissues and organs [20]. An ACh hydrolyzing enzyme, the acetylcholinesterase (AChE), is widely found in plants [21]. One of scarce available references [22], that reported the profiling of Salicaceae bud extracts through high-performance thin-layer chromatography, revealed the assignment of chrysin as a selective cholinesterase inhibitor. Transferred to sweet cherry buds, this means, that as long the chrysin content is high and stable, AChE is inhibited and a high content of ACh can maintained. The AChE catalyzes the hydrolysis of acetylcholine, and therefore this chemical reaction involves water. The ester group of acetylcholine is especially vulnerable to hydrolysis by water. The cleaving of ACh by AChE forms an alcohol (choline) and an acid (acetic acid). This can take place when the water content in the buds rises from ~60% at SB, continuously up to ~80% at OC, while the chrysin content decreases linearly and lost the function as AChE inhibitor. Therefore, chrysin as well the water content, or their interplay, can be suitable indicators for t_1_* in sweet cherry buds, whereby the determination of the water content in the buds is much easier than the analysis of chrysin. Therefore, the water content remains a reliable and practicable indicator for the beginning of ontogenetic development (t_1_*) in sweet cherry buds. 

### 3.2. Arabonic Acid in Sweet Cherry Buds

**Figure 2:**
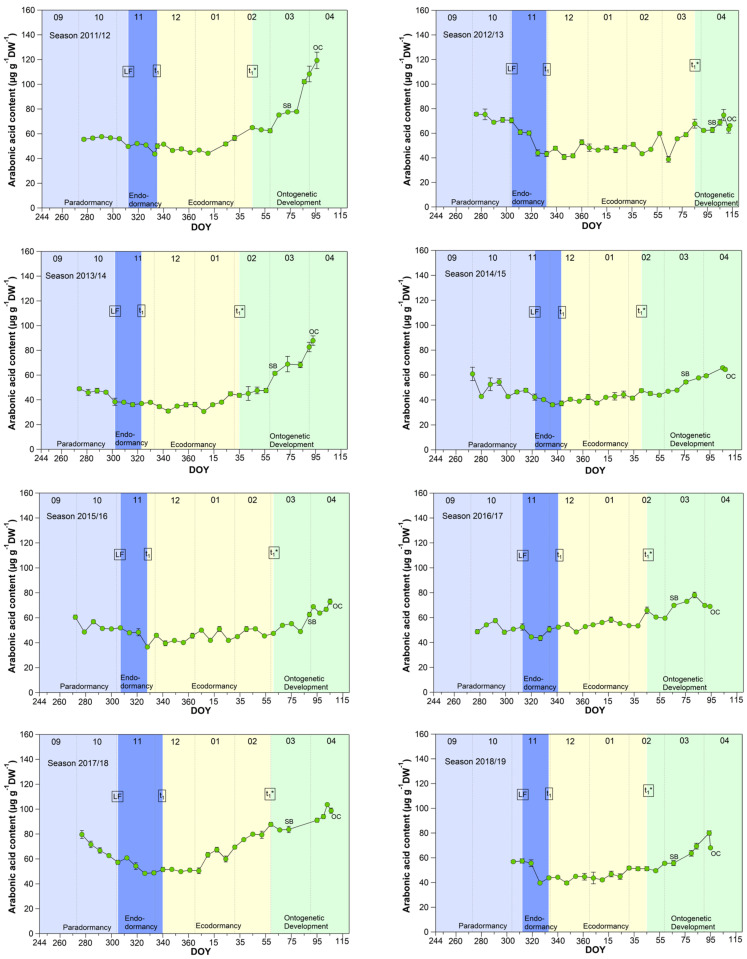

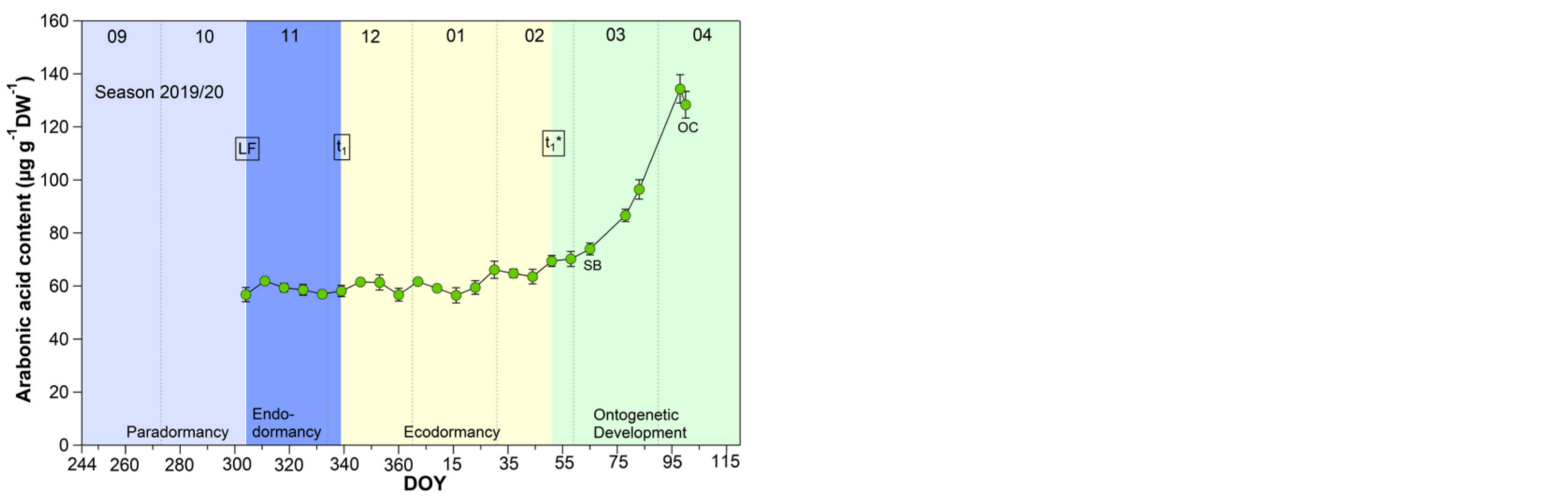
Arabonic acid in sweet cherry buds cv. Summit, 2011/12–2019/20. ‘Total leaf fall’ (LF), endodormancy release (t_1_), beginning of ontogenetic development (t_1_*), ‘swollen bud’ (SB) and ‘open cluster’ (OC).

The pattern for arabonic acid, identified as an ‘u-shape’ by the untargeted metabolite profiling 2015/2016 (scaled intensity) was in this form not always visible during the nine years sampled, but in all years a clear increase of arabonic acid at t_1_* was confirmed (Figure 2). 

Arabonic acid (Figure 2), a crystalline acid [23], is a reaction product which can be obtained by oxidation of arabinose (five-carbon-sugar, pentose), glucose, or fructose, (both six-carbon-sugars) and is a source for synthesizing riboflavin. Arabinose, for example, is involved in many different processes in plants, like cell wall flexibility, cell plate formation, cell-cell attachment, cell wall cross linking, desiccation resistance and signaling. Embryos and cotyledons of seeds contain the highest content of arabinose, compared to other plant tissue, whereby pollen grains contain ~40% in their cell walls [24].

Pentose phosphate pathway (PPP) constitutes an important reservoir for the building elements to enables plant cells to provide carbon skeleton for aromatic amino acids, phenolic compounds, and pentose sugars. Further, it is assumed that the PPP and their intermediates is involved in restraining and release, respectively, of differentiation of plant organs, and therefore in the embryogenesis [25]. 

According to the analyses for arabonic acid over 9 years, its course is mostly an indicator of t_1_*. Only in four years (2012/13, 2014/15, 2017/18, 2018/19) was a distinct reduction of the arabonic acid content between S1/LF and t_1_ confirmed. 

### 3.3. Pentose Acid in Sweet Cherry Buds

**Figure 3:**
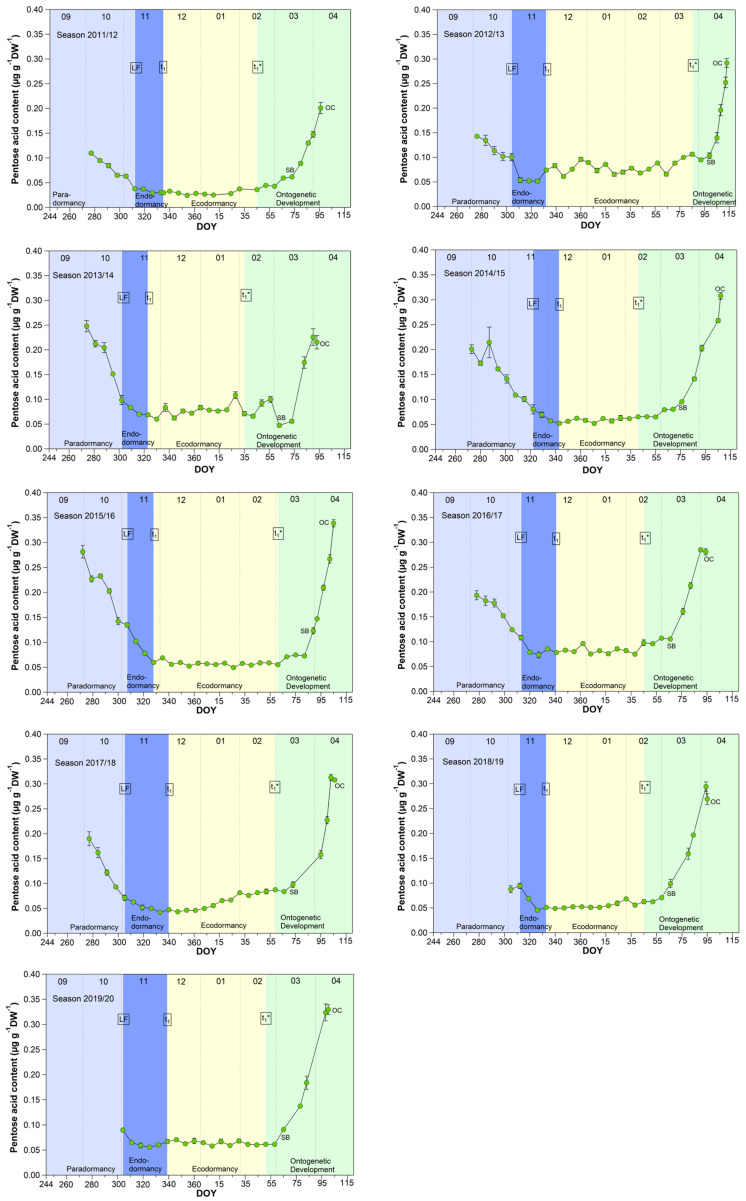
Pentose acid in sweet cherry buds cv. Summit, 2011/12–2019/20. ‘Total leaf fall’ (LF), endodormancy release (t_1_), beginning of ontogenetic development (t_1_*), ‘swollen bud’ (SB) and ‘open cluster’ (OC).

A signal, which corresponds to a 5-C sugar, was identified by METABOLON as “pentose acid”. The DNA and RNA nucleic acids hydrolysis can yield a pentose, whereby RNA is more susceptible to hydrolysis [26]. The pentose acid ideally describes the curve of winter rest, of the different dormancy phases, respectively, of the sweet cherry cv. Summit over the 9 years, with respect to metabolic activity (Figure 3). Initially, a strong decrease in the content occurred from the first sampling date (S1) until LF, which continues to weaken until t_1_. During ecodormancy (t_1_–t_1_*), pentose acid was maintained at a stable minimum of 0.065 ± 0.018 μg/(g DW). After t_1_*, but more pronounced after SB, the content significantly (*p* ≤ 0.05) increased from 0.092 (SB) to 0.282 μg/(g DW) at OC (Table 1). The RNA contains mostly hydrogen bonds, which are sensitive to changes in ambient temperature. The RNA is prone to hydrolysis at high temperatures and the appearance of stable RNA structures under cold environmental conditions may reduce reaction rates [26], shown as minimum pentose acid content during ecodormancy. 

From the physiological perspective, pentose acid in cherry buds would be a suitable metabolite to identify the non-observable stages t_1_ and t_1_*. However, the course of pentose acid would not allow to describe the ecodormancy phase in phenology models, because of its constantly low value during this phase in all years. Therefore, it only confirms the downregulation of biological activity during the main winter period, as described in [1].

### 3.4. Sucrose in Sweet Cherry Buds

**Figure 4:**
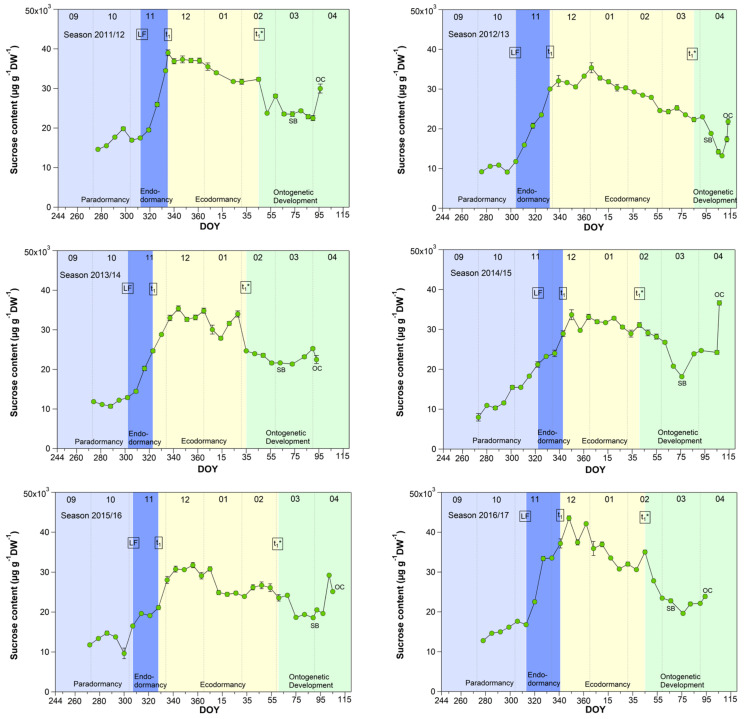

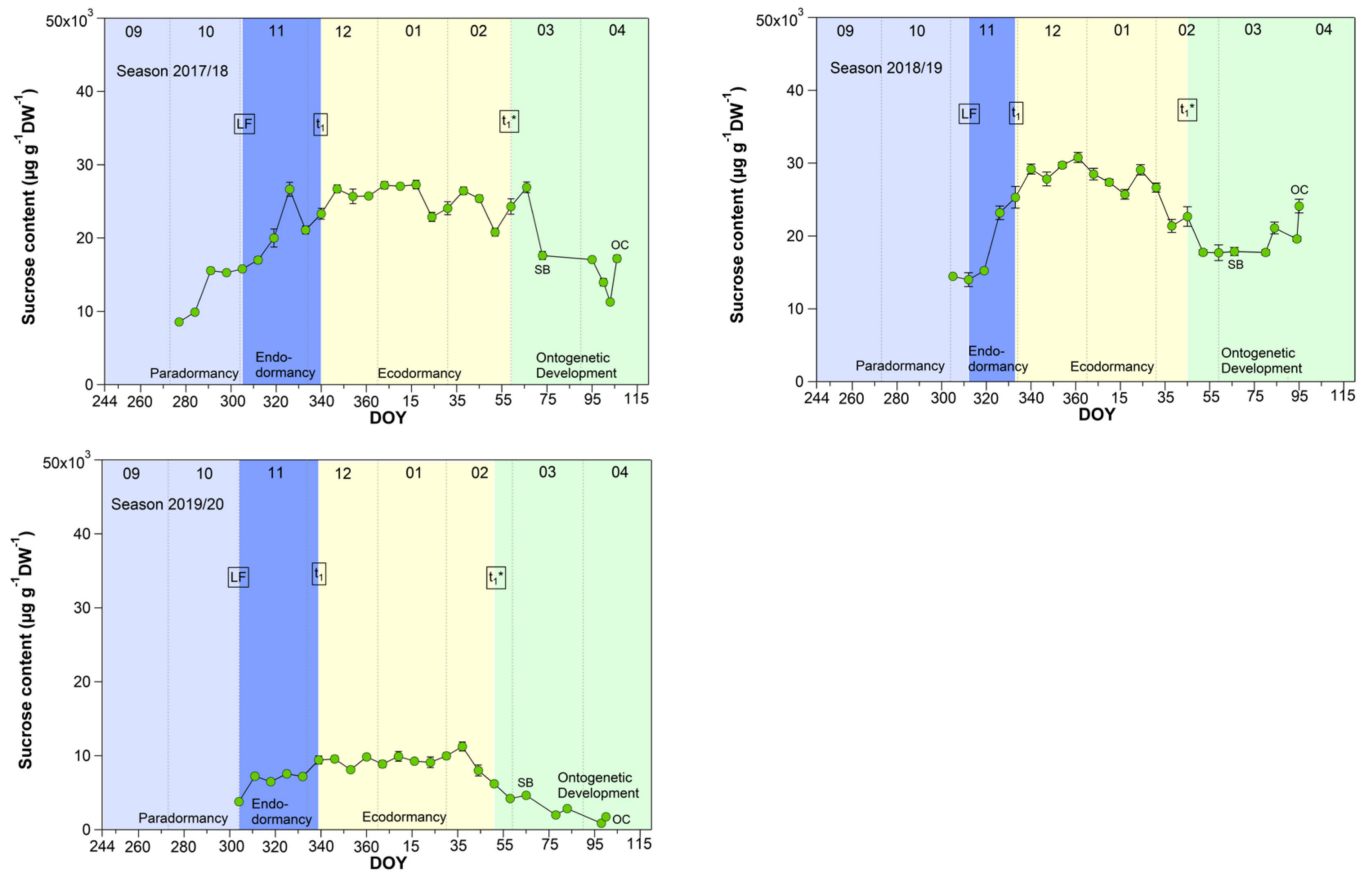
Sucrose in sweet cherry buds cv. Summit, 2011/12–2019/20. ‘Total leaf fall’ (LF), endodormancy release (t_1_), beginning of ontogenetic development (t_1_*), ‘swollen bud’ (SB) and ‘open cluster’ (OC).

As shown for five years [27], now also in a further four years, the sucrose content in buds strongly rose during the endodormancy phase (LF–t_1_) and reached its highest, nearly constant value during ecodormancy (Figure 4). With the beginning of ontogenetic development, it decreased nearly until the SB and subsequently rose again until the TC or OC phases. During nine years, the sucrose content started to increase stepwise from first sampling S1-LF, but mainly during endodormancy from 14.5 (LF) to 26.6 mg/(g DW) at t_1_. The highest mean sucrose content of 27.7 mg/(g DW) was observed during ecodormancy, ranging between 9.1 mg/(g DW) in 2019/20 and 35.8 mg/(g DW) in 2016/17. The low sucrose content in 2019/20 was related to a very mild winter. The average winter temperature in this year was 5.4 °C (mean 2.8 ± 1.5 °C), the mildest winter in nine years. As the result, we found a strong correlation between the mean air temperature and the sucrose content during ecodormancy (r = −0.81, *p* ≤ 0.01, n = 9), which confirms the cryoprotective role of sucrose in the flower buds but also in other plant organs, as already stated in [16,27,28,29].

### 3.5. Abscisic Acid Content in Sweet Cherry Buds

**Figure 5:**
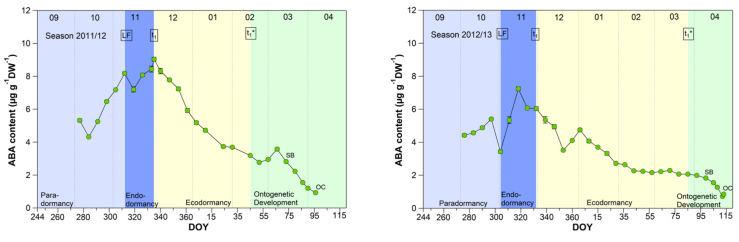

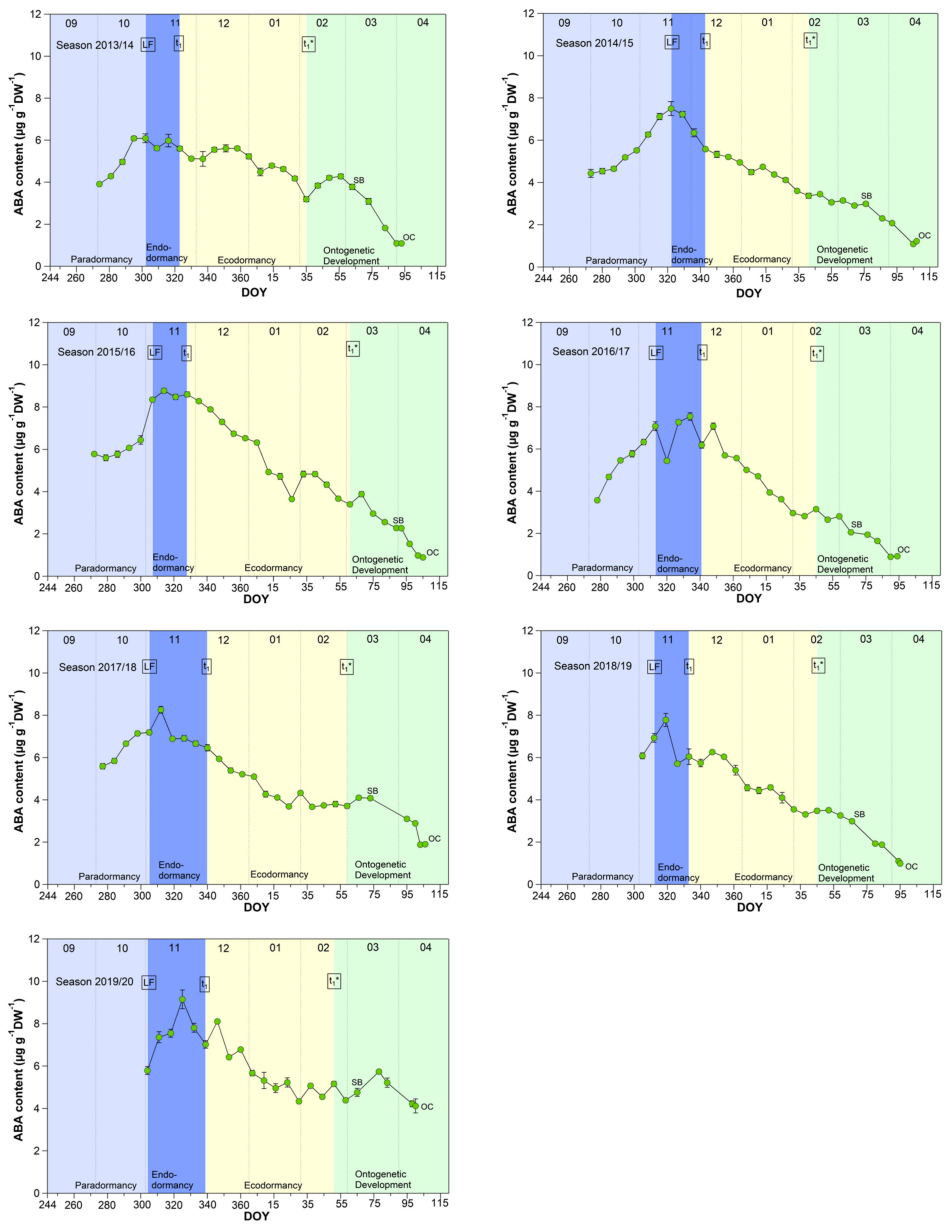
Abscisic acid in sweet cherry buds cv. Summit, 2011/12–2019/20. ‘Total leaf fall’ (LF), endodormancy release (t_1_), beginning of ontogenetic development (t_1_*), ‘swollen bud’ (SB) and ‘open cluster’ stage (OC).

Although abscisic acid only shows a clear change in the temporal course after the date of endodormancy release (Figure 5), it appears to be the key metabolite explaining t_1_ and t_1_* [2]. The physiological importance of ABA in the regulation of seed dormancy and seed development has been documented for decades [30,31,32,33,34], in the meantime, it is accepted that ABA during winter has a growth suppressing function in fruit trees. Physiological, metabolomic and transcriptomic studies have proposed the role for ABA in the inhibition of bud “activity” during winter rest [15,18]. Abscisic acid seems to have an important function in the establishment of endodormancy, as the blocking of cell-to-cell communication is mediated by ABA [35]. For sweet cherry cultivars, it has been reported that the ABA content is low before dormancy onset and shows a good match to the date of dormancy release [15]. Similar results were found for peach and pear [36,37]. 

The mean ABA content reached a significant (*p* ≤ 0.05, Table 1) maximum of 6.98 μg/(g DW) during the endodormancy phase and continuously decreased during ecodormancy, reaching on average 50% of the content of t_1_ at the beginning of ontogenetic development (t_1_*). The physiological importance of ABA to model cherry blossom has been described in detail in [2]. Consequently, the ABA content allows to define the date of endodormancy release, because after t_1_ the content continuously decreases until t_1_* and finally up to OC. 

### 3.6. Abscisic Acid Glucose Ester (ABA-GE) Content in Sweet Cherry Buds

**Figure 6:**
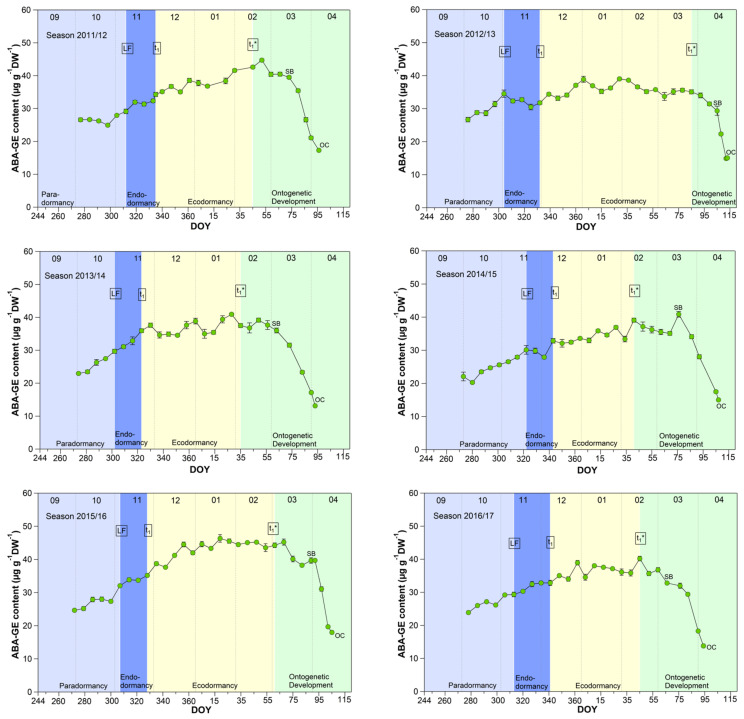

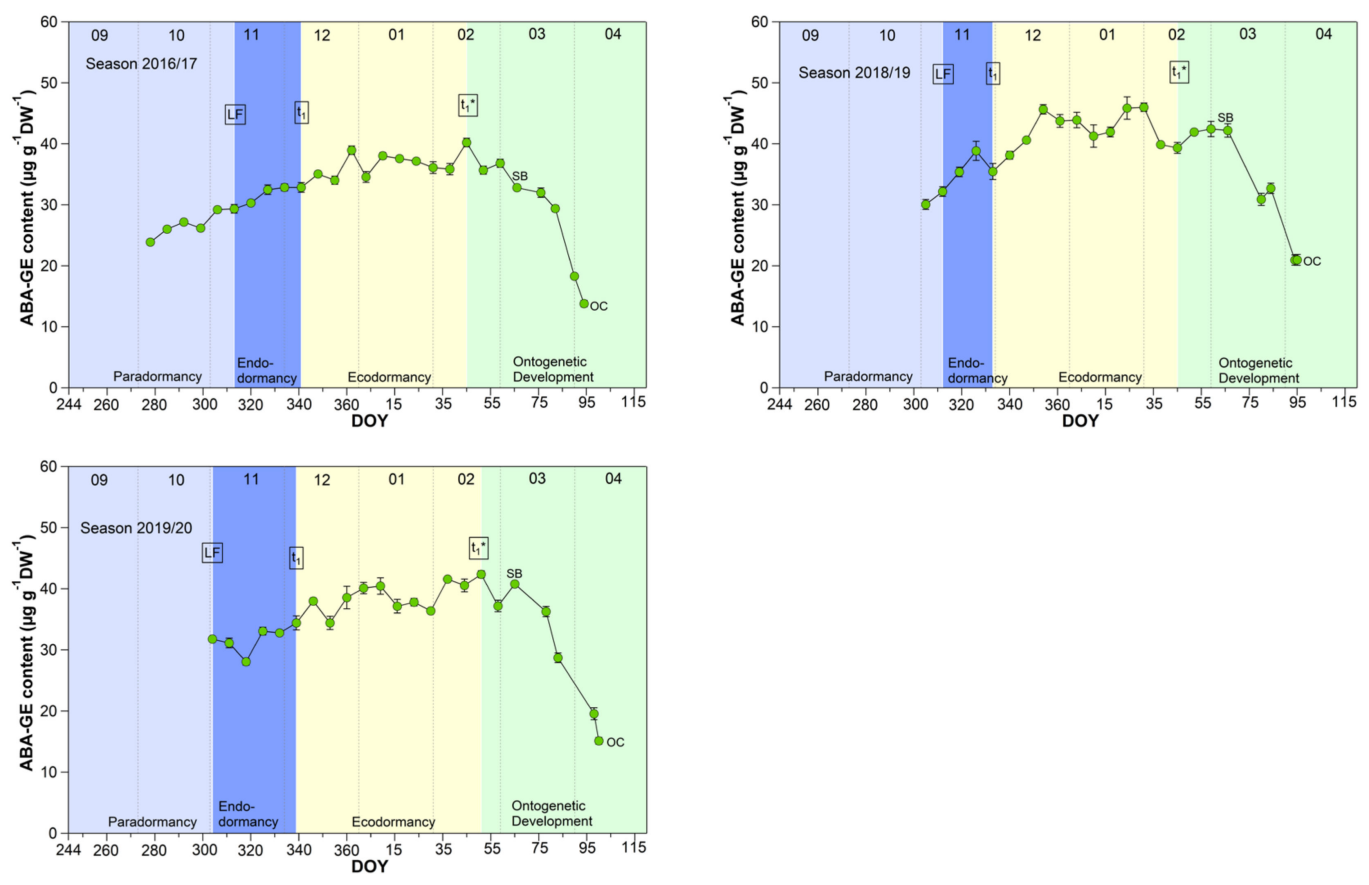
Abscisic acid glucose ester (ABA-GE) in sweet cherry buds cv. Summit, 2011/12–2019/20. ‘Total leaf fall’ (LF), endodormancy release (t_1_), beginning of ontogenetic development (t_1_*), ‘swollen bud’ (SB) and ‘open cluster’ stage (OC).

The mean ABA-GE content reached 33.4 μg/(g DW) during the endodormancy phase and increased significant (*p* ≤ 0.05) to a stable content of about 39.0 μg/(g DW) during ecodormancy and the phase t_1_*–SB (Table 1, Figure 6). In the phase SB-OC, the ABA-GE content decreased by ~36% to 24.6 μg/(g DW), the lowest content during observation. The mean ABA-GE content in the buds was at all phenological stages (LF, t_1_, t_1_*, SB, OC, respectively, 4.7, 5.2, 11.8, 12.5, 11.4-fold), as well as during endo-, ecodormancy phase and the ontogenetic development (LF-t_1_, t_1_–t_1_*, t_1_*-SB, SB-OC, respectively, 4.7, 8.3, 12.3, 12.6-fold) higher than the ABA content. Because hydrolysis of ABA-GE releases free ABA, ABA-GE can be considered as a readily available storage pool in cherry buds. 

Concerning ABA catabolism [14,38], three different hydroxylation pathways are documented. The hydroxylation triggers further inactivation steps, resulting in the corresponding formation of phaseic acid (PA) and dihydrophaseic acid (DPA). In addition to hydroxylation pathways, ABA can also be conjugated to glucose resulting in the formation of abscisic acid glucosyl ester (ABA-GE). The conjugation product ABA-GE is thought to be an inactive storage or transport form of ABA. Compared to the oxidative pathway, the inactivation of ABA by glucose conjugation is reversible, and hydrolysis of ABA-GE catalyzed by *beta*-glucosidases results in free ABA. These catabolites are important for ABA homeostasis, and on the other hand, emerging metabolites and intermediates and their resulting “signaling effects” can have controlling influence on the transitions of phenological phases [14].

## 4. Conclusions

For the development of physiologically based phenology models, it is essential that the sequence of metabolite production is available for several years, because multi-year data allow to evaluate the annual variability of the metabolite content and, if necessary, to relate it to environmental factors. The major challenge here is that the date of non-observable dates, t_1_ and t_1_*, cannot be recorded by phenological observations. It was necessary to detect the timing of t_1_ and t_1_* before it was possible to identify any metabolites that might be related to the induction or release of phenological phases. Metabolites which showed trend changes at the important stages, t_1_ and/or t_1_* can be key markers which confirm the previously found dates for t_1_ and t_1_*. However, the course of these metabolites would not allow to describe the ecodormancy phase in phenology models, because of its constantly low (arabonic acid, pentose acid) or high (chrysin, sucrose, abscisic acid glucose ester) content during this phase. Consequently, they only confirm the up- or downregulation of biological activity in buds during the main winter period. Although ABA only shows a clear change in the temporal course after the date of endodormancy release, it appears to be the key metabolite explaining t_1_ and t_1_*, and therefore the ecodormancy phase, which must be better considered in phenology models.

## Figures and Tables

**Table 1:** Average content (mean of nine years ± standard deviation) of six selected metabolites in μg/(g DW) at five phenological stages ‘total leaf fall’ (LF), endodormancy release (t_1_), beginning of ontogenetic development (t_1_*), ‘swollen bud’ (SB), ‘open cluster’ (OC) as well as during four phenological phases: endo- (LF–t_1_), ecodormancy phase (t_1_–t_1_*) and ontogenetic development (t_1_*–SB; SB–OC). Different letters indicate significant differences between the metabolite content within stages and phases (ANOVA, Tukey-HSD test, *p* ≤ 0.05).

Metabolite	LF	t_1_	t_1_*	SB	OC	LF–t_1_	t_1_–t_1_*	t_1_*–SB	SB–OC
Chrysin	186.4 ^b^ ± 45.97	194.5 ^b^ ± 39.90	196.0 ^b^ ± 38.62	185.7 ^b^ ± 38.69	76.2 ^a^ ± 37.93	194.0 ^b^ ± 41.88	194.1 ^b^ ± 40.66	192.3 ^b^ ± 34.68	110.1 ^a^ ± 50.59
Arabonic acid	53.0 ^ab^ ± 9.33	45.5 ^a^ ± 7.82	60.6 ^ab^ ± 14.30	66.9 ^bc^ ± 10.02	86.1 ^c^ ± 24.26	49.5 ^a^ ± 8.55	50.4 ^a^ ± 10.72	59.5 ^a^ ± 11.59	80.9 ^b^ ± 19.55
Pentose acid	0.090 ^a^ ± 0.027	0.059 ^a^ ± 0.015	0.071 ^a^ ± 0.022	0.092 ^a^ ± 0.023	0.282 ^b^ ± 0.048	0.066 ^a^ ± 0.023	0.065 ^a^ ± 0.018	0.080 ^a^ ± 0.021	0.218 ^b^ ± 0.073
Sucrose	14,476.2 ^a^ ± 4874.0	26,552.8 ^b^ ± 8839.2	24,691.8 ^ab^ ± 8361.5	18,184.3 ^ab^ ± 5544.1	22,555.1 ^ab^ ± 9530.5	20,260.5 ^a^ ± 8348.3	27,688.8 ^b^ ± 7730.1	21,249.5 ^a^ ± 5856.5	19,438.3 ^a^ ± 7919.0
Abscisic acid	6.73 ^c^ ± 1.49	6.73 ^c^ ± 1.27	3.41 ^b^ ± 0.80	3.06 ^b^ ± 0.98	1.43 ^a^ ± 1.06	6.98 ^a^ ± 1.20	4.67 ^b^ ± 1.41	3.24 ^c^ ± 0.76	1.95 ^d^ ± 1.23
Abscisic acid glucose ester	31.6 ^b^ ± 2.27	34.9 ^bc^ ± 2.86	40.3 ^d^ ± 2.85	38.3 ^cd^ ± 3.91	16.2 ^a^ ± 2.43	33.4 ^b^ ± 3.40	39.0 ^c^ ± 3.84	38.9 ^c^ ± 3.96	24.6 ^a^ ± 8.12

## Data Availability

Data is not publicly available due to privacy or ethical restrictions. Output of the targeted metabolite identification is available on reasonable request to the corresponding author.

## Supplementary Materials

The following supporting information can be downloaded at https://www.mdpi.com/article/10.3390/metabo13020231/s1. Figure S1: Chemical structure of (a) chrysin, (b) abscisic acid, and (c) abscisic acid glucosyl ester (ABA-GE).
