# Supplementary material for: Metabolites That Confirm Induction and Release of Dormancy Phases in Sweet Cherry Buds
Source: Metabolites. 2023 Feb 3;13(2):231. doi: 10.3390/metabo13020231 (PMC9961560; doi:10.3390/metabo13020231)
Supplement: Supplementary file 1 [file metabolites-13-00231-s001.zip › Supplemental Materials Figure S1.pdf]

Figure S1. Chemical structure of (a) chrysin, (b) abscisic acid and (c) abscisic acid glucosyl ester (ABA-GE).

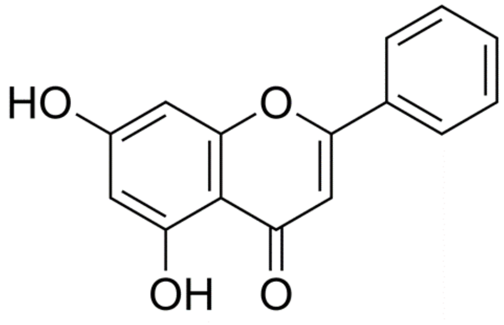

(a) Chemical structure of 5,7-dihydroxyflavone (chrysin)

Source: https://doi.org/10.1016/j.neuint.2020.104850

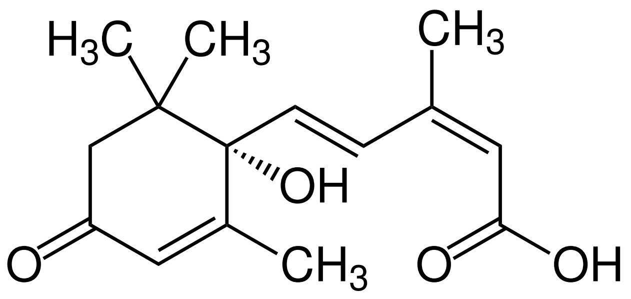

(b) Chemical structure of abscisic acid (ABA)

Source: https://www.sigmaaldrich.com

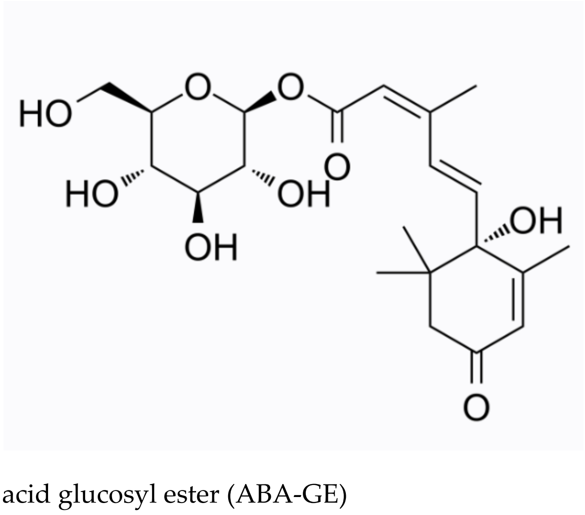

(c) (S)-*cis,trans*-Abscisic acid glucosyl ester (ABA-GE)

Source: https://www.medchemexpress.com
